# Severe G6PD deficiency leads to recurrent infections and defects in ROS production: Case report and literature review

**DOI:** 10.3389/fgene.2022.1035673

**Published:** 2022-10-24

**Authors:** Bijun Sun, Qifan Li, Xiaolong Dong, Jia Hou, Wenjie Wang, Wenjing Ying, Xiaoying Hui, Qinhua Zhou, Haili Yao, Jinqiao Sun, Xiaochuan Wang

**Affiliations:** ^1^ Department of Clinical Immunology, Children’s Hospital of Fudan University, Shanghai, China; ^2^ Shanghai Institute of Infectious Disease and Biosecurity, Shanghai, China

**Keywords:** G6PD gene, infection, ROS, chromosome inactivation, NF-κB pathway

## Abstract

**Purpose:** Severe glucose-6-phosphate dehydrogenase (G6PD) deficiency can lead to reduced nicotinamide adenine dinucleotide phosphate oxidase activity in phagocytes, resulting in immunodeficiency, with a limited number of reported cases. Here, we aimed to report a child with severe G6PD deficiency in China and investigate the mechanism of his recurrent infections.

**Methods:** The clinical manifestations and immunological phenotypes of this patient were retrospectively collected. Gene mutation was detected by whole-exome sequencing and confirmed by Sanger sequencing. Dihydrorhodamine (DHR) analysis was performed to measure the respiratory burst of neutrophils. Messenger ribonucleic acid and protein levels were detected in the patient under lipopolysaccharide stimulation by real-time quantitative reverse transcription polymerase chain reaction and Western blot. A review of the literature was performed.

**Results:** A male child with G6PD deficiency presented with recurrent respiratory infections, Epstein‒Barr virus infection and tonsillitis from 8 months of age. Gene testing revealed that the proband had one hemizygous mutation in the *G6PD* gene (c.496 C>T, p. R166C), inherited from his mother. This mutation might affect hydrophobic binding, and the G6PD enzyme activity of the patient was 0. The stimulation indexes of the neutrophils in the patient and mother were 22 and 37, respectively. Compared with healthy controls, decreased reactive oxygen species (ROS) production was observed in the patient. Activation of nuclear factor kappa-B (NF-κB) signaling was found to be influenced, and the synthesis of tumor necrosis factor alpha (TNF-α) was downregulated in the patient-derived cells. In neutrophils of his mother, 74.71% of the X chromosome carrying the mutated gene was inactivated. By performing a systematic literature review, an additional 15 patients with severe G6PD deficiency and recurrent infections were identified. Four other *G6PD* gene mutations have been reported, including c.1157T>A, c.180_182del, c.514C>T, and c.953_976del.

**Conclusion:** Severe G6PD deficiency, not only class I but also class II, can contribute to a chronic granulomatous disease-like phenotype. Decreased reactive oxygen species synthesis led to decreased activation of the NF-κB pathway in G6PD-deficient patients. Children with severe G6PD deficiency should be aware of immunodeficiency disease, and the DHR assay is recommended to evaluate neutrophil function for early identification.

## Introduction

Glucose-6-phosphate dehydrogenase (G6PD) deficiency is one of the most common clinically significant enzymopathies and affects 400 million persons worldwide (Nkhoma et al., 2009). Based on the level of residual enzyme activity, the World Health Organization (WHO) has categorized G6PD deficiencies into five classes: I (<10% of normal, chronic nonspherocytic hemolytic anemia); II (<10% of normal); III (10–60% of normal); IV (60–150% of normal), and V (>150% of normal) (WHO Working Group, 1989). A wide range of biochemical and clinical phenotypes have been observed. G6PD plays an important role in protecting red blood cells from oxidative stress, and the most common clinical manifestations of G6PD-deficient patients are neonatal jaundice and acute hemolytic anemia (Ryan and Tekwani, 2021).

Current diagnostic methods of G6PD deficiency include enzyme activity testing and gene sequencing (Shen et al., 2022; Xia et al., 2022). The G6PD gene encoding G6PD is located on the X chromosome, so this disease is inherited in the dominant mode of the X chromosome. Males with this condition are always hemizygous, while females may be heterozygous (He et al., 2020). More than 200 mutations of G6PD have been reported. G6PD hotspot mutations in the Chinese population include His32Arg, Gly131Val, Gly163Ser, Asn165Asp, Arg198Cys, Leu342Phe, Arg454Cys, Arg459Leu and Arg463His (Yan et al., 2010). Neonates are often routinely screened for G6PD deficiency by detecting G6PD enzyme activity with a rapid fluorescent spot test (Lee et al., 2022). The G6PD enzyme activity of neonatal male patients was mainly class II and III, while the enzyme activity of female heterozygous carriers was generally class IV (Chen et al., 2018).

G6PD is a key rate-limiting enzyme catalyzing the conversion of glucose 6-phosphate into 6-phosphogluconolactone (Khan et al., 2017). This is the first step in the pentose phosphate pathway. It provides reducing power to all cells through the production of coenzyme nicotinamide adenine dinucleotide phosphate (NADPH) (Nóbrega-Pereira et al., 2016). G6PD deficiency is typically restricted to red blood cells, in which the pentose phosphate pathway is the unique source of NADPH (Efferth et al., 2004). Therefore, G6PD deficiency is usually considered a disease that leaves red blood cells vulnerable to oxidative damage.

Severe loss of enzyme activity can lead to NADPH oxidase deficiency in phagocytes. NADPH is essential for the generation of reactive oxygen species (ROS), which are required for the microbicidal activity of phagocytes through the phagocytic NADPH oxidase complex (Zhang et al., 2022). As an important cellular signaling molecule, ROS are involved in a variety of intracellular activities, including the initiation of the nuclear factor kappa-B (NF-κB) pathway (Sies et al., 2022). Chronic granulomatous disease (CGD) is a disease characterized by defective respiratory burst function and recurrent infections (Yu et al., 2021). Severely impaired G6PD activity can also lead to decreased NADPH oxidase activity, resulting in decreased ROS production (Siler et al., 2017) and CGD-like symptoms in patients. G6PD deficiency class I has already been listed as a primary immunodeficiency disease (PID) (Picard et al., 2018).

Although phagocytes from most G6PD-deficient patients have normal bactericidal activity, rare cases with severe G6PD deficiency (van Bruggen et al., 2002) have been reported to be prone to infection. The understanding of this subset of patients is still insufficient and should not be ignored by clinicians. In this study, we report a Chinese child with severe G6PD deficiency presenting with CGD-like symptoms and explore ROS production, activation of the NF-κB pathway and the expression of related inflammatory cytokines. Then, we systematically reviewed the literature to further deepen our understanding of severe G6PD deficiency.

## Materials and methods

The study was approved by the Ethics Committee of the Children’s Hospital of Fudan University (No. 2019 017) on 27 February 2019. The patient and his parents provided written informed consent for enrollment in this study.

### Patient and controls

A child who had G6PD deficiency with recurrent infections was included in this study. His clinical manifestations, immunological features and genetic sequencing were retrospectively summarized. Three healthy volunteers were recruited as controls for the subsequent trial, including ROS production, real-time quantitative reverse transcription polymerase chain reaction (PCR), and western blot.

### Dihydrorhodamine analysis

A dihydrorhodamine (DHR) assay was performed to measure the respiratory burst of neutrophils by testing superoxide production. Briefly, neutrophils were stimulated with 10 μg/ml phorbol-12-myristate-14-acetate (PMA) (Sigma‒Aldrich, United States), followed by incubation with dihydrorhodamine-1,2,3 (Sigma‒Aldrich, United States). Flow cytometric analysis was performed as previously described by using a FACS Calibur flow cytometer (Becton Dickinson, Franklin Lakes, NJ, United States). The stimulation index (SI) was defined as the geometric fluorescence intensity of PMA-stimulated neutrophils over the geometric fluorescence intensity of unstimulated neutrophils.

### Genetic analysis

Genomic deoxyribonucleic acid (DNA) was extracted from the peripheral blood of the patient and his parents using the QIAamp DNA Blood Mini kit (Qiagen, Hilden, Germany). DNA quality was assessed using a NanoDrop ultraviolet spectrophotometer (Thermo Fisher Scientific, United States). Whole-exome sequencing (WES) and analysis protocols were adapted for genetic analysis. Enriched DNA samples were indexed and sequenced on the HiSeq 2000 platform (Illumina, San Diego, CA) in accordance with the manufacturer’s instructions. Nucleotide changes observed in more than 5% of aligned reads were identified and reviewed using NextGENe software (SoftGenetics, State College, PA). Sanger sequencing was performed to confirm the mutation of *G6PD* (Dong et al., 2019). The impact of the missense mutation on protein function and structure was assessed by PyMOL software (Schrödinger, United States).

### Peripheral blood mononuclear cell isolation and cell culture

Venous blood was drawn from this patient, his mother and three healthy volunteers. An equal volume of phosphate buffered saline (PBS) (Meilunbio, Dalian, China) was added to ethylene diamine tetraacetic acid (EDTA) anticoagulated blood. The diluted blood was carefully added to the top of Ficoll-Paque Plus media (Amersham Pharmacia Biotech, Baie-D'Urfé, Quebec, Canada) in a centrifuge tube (Corning, NY, United States) and centrifuged at 2,000 rpm for 20 min at room temperature. After being washed twice in PBS, the peripheral blood mononuclear cells (PBMCs) were cultured in RPMI 1640 (Sigma‒Aldrich, United States) supplemented with 10% fetal bovine serum (FBS) (Sigma‒Aldrich, United States) at a density of 1×10^6^ cells/ml. Ultrapure *Escherichia coli* lipopolysaccharide (LPS) (Sigma‒Aldrich, United States) was added to the cell culture at a final concentration of 100 ng/ml for stimulation. After incubation in a 12-well plate at 37°C in 5% CO_2_ for 24 h, the cells were harvested for subsequent experiments.

### Reactive oxygen species detection

ROS production in PBMCs was tested by treatment with the fluorescent probe 2′,7′-dichlorofluorescein diacetate (DCFH-DA) after LPS stimulation. Intracellular ROS production in PBMCs was detected by a reactive oxygen species assay kit (Beyotime Biotechnology, Haimen, China) following the manufacturer’s instructions. A total of 1×10^6^ cells per well were seeded in a 24-well plate and stimulated with LPS for 2 h. After stimulation, the cells were washed twice with PBS and then cultured with 10 μM of the DCFH-DA in serum-free RPMI-1640 medium (Sigma‒Aldrich, United States) for 20 min. After being washed 3 times, the cells were resuspended in 1 ml serum-free medium. DCF fluorescence intensity was detected at an excitation wavelength of 488 nm to quantify the levels of ROS.

### Ribonucleic acid extraction

Total ribonucleic acid (RNA) was extracted using RNAiso Plus Reagent (TaKaRa, Japan) following the manufacturer’s instructions.

### Real-time quantitative reverse transcription polymerase chain reaction

Complementary DNA (cDNA) was synthesized using Transcript RT Master Mix (Takara, Japan). Real-time quantitative reverse transcription PCR was performed to detect transcript expression using Takara SYBR Fast qPCR Mix (Takara, Japan) on a LightCycler 480 Instrument II (Roche, Switzerland) (Wang et al., 2018; Marfella et al., 2022). The primer sequences were as follows: G6PD (Forward): 5′-AGC​TGG​ACT​TCT​TTG​CC-3′, G6PD (Reverse): 5′-TGA​TGC​GGT​TCC​AGC​CTA​TC-3′, tumor necrosis factor (TNF)-α (Forward): 5′-TCT​CTC​CCC​TGG​AAA​GGA​CA-3′, TNF-α (Reverse): 5′-AGA​GGC​TGA​GGA​ACA​AGC​AC-3′. Actin (Forward): 5′-GCA​AAG​ACC​TGT​ACG​CCA​AC-3′, Actin (Reverse): 5′-CAT​CTG​CTG​GAA​GGT​GGA​CA-3′. The reactions included 2 μl of cDNA, 10 μl of SYBR Green Mix, 0.8 μl each of forward and reverse primers and 6.4 μl of ddH_2_O. The thermocycler conditions were 95°C for 30 s, followed by 40 cycles at 95°C for 5 s, 60°C for 20 s and 72°C for 20 s. A melting curve analysis was performed at the end of the expression analysis using the following conditions: 55°C for 60 s, followed by 81 cycles starting at 55°C for 10 s with a 0.5°C increase with each cycle. Relative expression data were obtained using the 2^−ΔΔCT^ method. The β-actin gene was used as an internal reference, and the expression of the target gene was quantified relative to the mRNA levels in unstimulated cells from healthy controls. The experiments were performed with three replicates.

### Western blot

In the present study, the influence of the *G6PD* mutation was assessed by performing immunoblotting. Western blotting was performed as previously described (Dong et al., 2019; Li et al., 2022; Wang et al., 2022). Cytoplasmic and nuclear protein extraction was performed using NE-PER™ Nuclear and Cytoplasmic Extraction Reagents (Thermo Fisher Scientific, United States). Equal amounts of cytoplasmic or nuclear extracts were separated on 12% SDS polyacrylamide gels and transferred to PVDF membranes. Blots were probed with primary antibodies against NF-κB p65, β-actin or histone H3 (Cell Signaling Technology, Beverly, MA). Primary antibodies were detected with horseradish peroxidase-conjugated secondary antibody. Visualization was conducted using an ECL peroxidase substrate (Millipore, Germany). The molecular weight of NF-κB p65 and p-p65 is 65 kD, β-actin is 45 kD, and histone H3 is 17 kD.

### Hpa II digestion and X chromosome inactivation detection

Hpa-II enzyme digestion was used to detect the ratio of inactivated X-chromosome carrying the mutated *G6PD* sequence. Maternal peripheral blood and neutrophil DNA were extracted. The reactions included 2 μl of cDNA, 1 μl of Hpa II, 2 μl of NEBuffer and 15 μl of ddH2O. After digestion by Hpa II (NEB, Ipswich, MA) at 37°C for 16 h, the reaction was terminated after incubation at 95°C for 10 min. DNA samples from healthy men were used as controls to assess the completeness of the reaction. PCR amplification was performed, and the two X chromosome peaks after Hpa digestion were compared by GeneMarker V2.9.0 (BioGene Ltd, United Kingdom). The X chromosome inactivation rate was calculated as (d1/u1)/(d1/u1+d2/u2). d1 and d2 represent the peak values of two bands of PCR products of enzyme-cut samples, and u1 and u2 represent the two band peaks of PCR products of undigested samples.

### Literature review

This review was conducted on case series that focused on G6PD deficiency with neutrophil dysfunction. The eligible articles published until 2022 were searched in PubMed. The search process was performed by two researchers who were continuously in contact with each other to exchange information by searching PubMed up to 18 September 2022. The search process was accomplished using the following keywords: “Neutrophils” or “Leukocytes” and “Glucosephosphate Dehydrogenase Deficiency”. The inclusion criteria were as follows: (i) clear diagnosis of G6PD deficiency and (ii) clinical manifestations of infection or clear etiological diagnosis. The exclusion criteria were (i) publication in languages other than English, (ii) normal bactericidal activity, (iii) insufficiency of the presented data, and (iv) phagocyte dysfunction caused by other causes, such as PID. In addition, cross-sectional investigations, experimental studies, and systematic reviews were removed from the review process.

First, the titles and abstracts of all papers were studied, and the articles related to the subject of interest were selected. Then, the full texts of the selected papers were obtained, and the articles appearing to meet the inclusion criteria were reviewed. The extraction of the information was accomplished using a predesigned data extraction form. According to the purpose of our study, the risk of bias is not applicable.

The related data, such as age, sex, clinical features, pathogens, antibiotic therapy, G6PD activity, neutrophil function tests, changes in genetics, and outcomes, were extracted from all the included papers. The selection process of the papers is shown in a PRISMA flow diagram.

## Results

### Case report: Clinical manifestations

This male patient, gravida 1 para 1, was born to nonconsanguineous Chinese parents. At 8 months of age, he was admitted to our hospital due to influenza and bronchopneumonia. The child weighed 8.5 kg and had a body mass index (BMI) of 15.7. The patient presented with a fever again 2 months later. He had Epstein‒Barr virus (EBV) infections confirmed by EBV DNA detection and then recovered through effective treatment with acyclovir. The patient suffered from purulent tonsillitis and adenoviral enteritis accompanied by abnormal hepatic function at the age of 18 months. He developed recurrent cough and fever 1 week after discharge. Chest CT examination showed pneumonia, but no pathogen had been cultured. Sheet maculopapules were seen in the thigh and scrotum during hospitalization, and cutaneous candidiasis was considered. The child received anti-infective therapy of intravenous cephalosporin and oral itraconazole. Recurrent fever, sore throat, and tonsillitis have occurred approximately 5–6 times a year since the age of 2 years, and the child is now 5 years old.

In addition to infection, this patient had other clinical manifestations. Dilatation of the intestine was detected by ultrasonography at the 28th week of gestation. The result of antenatal chromosomal screening appeared to be normal. Abdominal distension was evident after delivery such that ileal atresia was found by laparoscopic exploration. Eleven months after the operation, the child developed adhesive intestinal obstruction with effective anti-infection treatment. He was found to have hearing loss at 3 months old. His hearing screening suggested extremely severe binaural deafness. A history of recurrent eczema was elicited. The child had moderate anemia at 2 years of age and no acute hemolytic crisis.

### Immunological features

**FIGURE 1:**
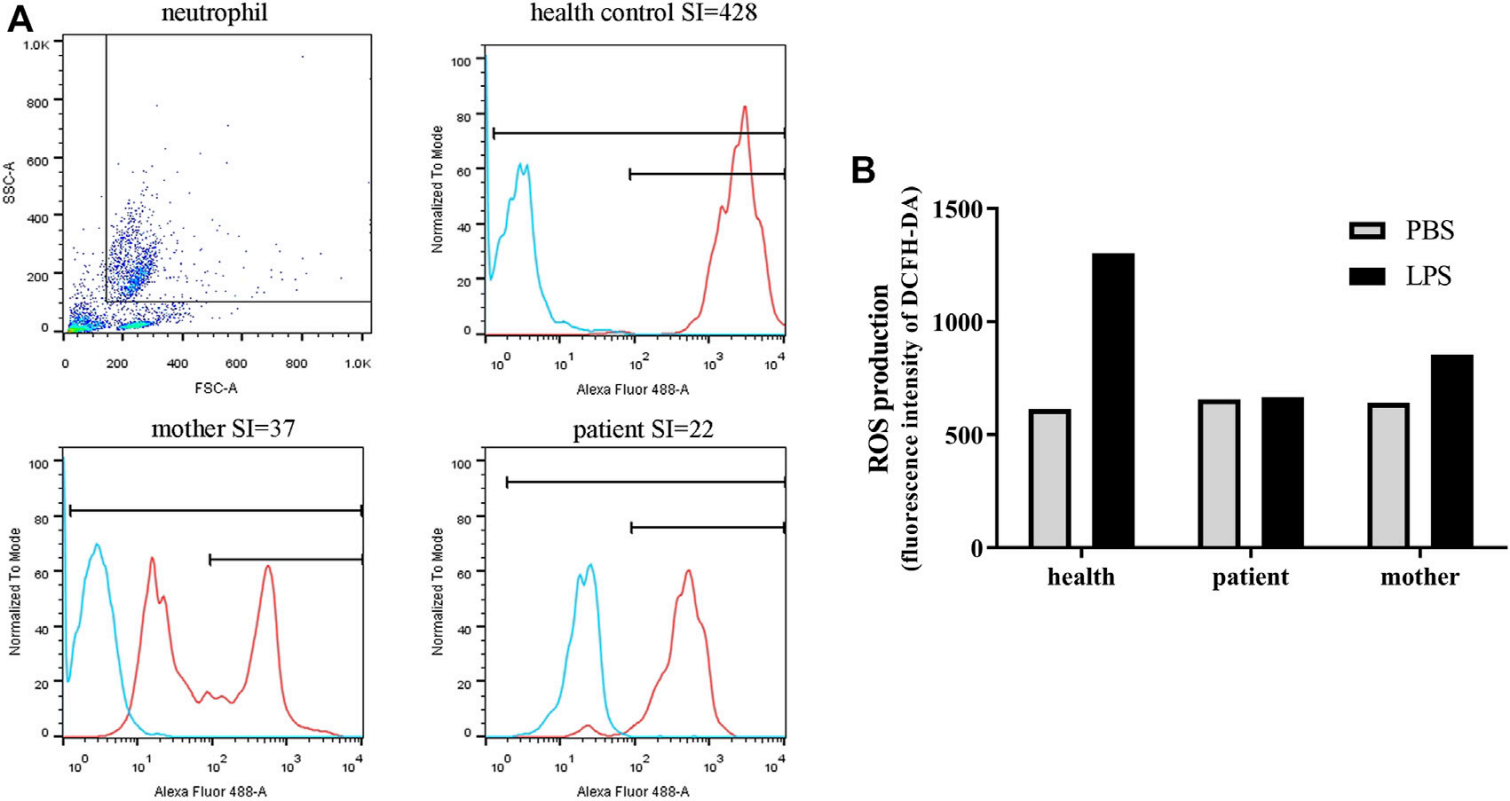
Neutrophil function and ROS production of the patient and his mother. **(A)** The SI values (neutrophil function) measured by DHR analysis with flow cytometry. Blue curve, stimulated with PBS; red curve, stimulated with PMA. **(B)** ROS production in PBMCs as measured by DCFH-DA. After stimulation, ROS production was obviously enhanced in healthy PBMCs, whereas no difference in fluorescence intensity was observed in the patient cells.

Due to recurrent respiratory infections, his immunological indicators were further evaluated. No obvious abnormality was detected in immunoglobulin and lymphocyte subsets (Table 1). Respiratory burst activity assessed by the DHR assay showed that the SIs of the neutrophils in the patient and mother were 22 and 37, respectively (Figure 1A). In the healthy control, the value of SI was 428. In addition, the DHR assay in the carrier mother showed bimodal curves after stimulation, indicating the existence of two granulocyte populations. This patient was initially suspected to have X-linked CGD at 1 year old.

**TABLE 1:** Distribution of the lymphocyte subsets and immunoglobulin in the patient.

	Patient	References
CD3 (cells/ul)	2046.0 (54.52%)	1794–4247
CD4 (cells/ul)	1186.16 (31.61%)	902–2253
CD8 (cells/ul)	782.15 (20.84%)	580–1735
CD19 (cells/ul)	808.03 (21.53%)	461–1456
CD16CD56 (cells/ul)	846.85 (22.57%)	270–1053
IgG (g/L)	4.50	3.50–8.90
IgM (g/L)	0.20	0.36–1.20
IgA (g/L)	0.21	0.06–0.54
IgE (KU/L)	6.62	<100
C3 (g/L)	1.20	0.67–1.76
C4 (g/L)	0.38	0.1–0.4
CH50 (U/ml)	40	23–46

Ig, immunoglobulins; C, complement

### Genetic analysis

**FIGURE 2:**
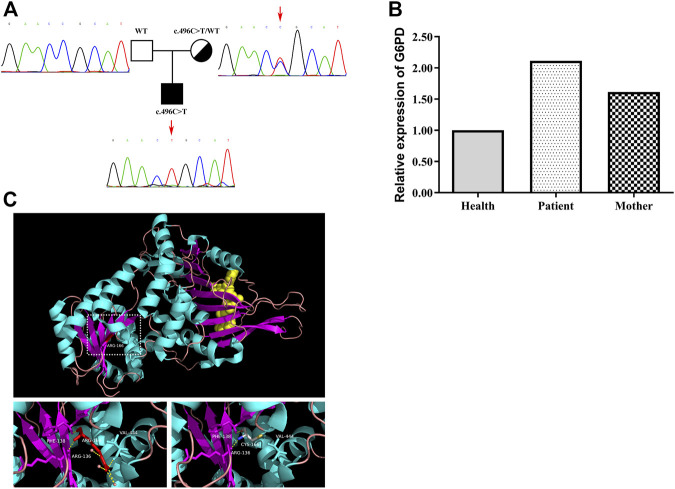
Gene analysis of the G6PD mutation in the patient and his mother. **(A)** Family tree and confirmation of mutation c.496 C>T in G6PD by Sanger sequencing. **(B)** Level of G6PD mRNA in PBMCs derived from the healthy control, the patient and the mother. The mRNA level of G6PD was higher in the patient than in the healthy control and mother. **(C)** Schematic model of WT and the mutated G6PD protein. The p. R166C mutant (right) might affect the hydrophobic binding of the amino acid residues to other nearby residues.

At the age of 14 months, the child was confirmed to have PID combined with clinical manifestations, immune indicators and gene analysis. The WES result revealed that he had one mutation in the *G6PD* gene (c.496 C>T, p. R166C), inherited from his mother. No mutations were detected in other immunodeficiency-related genes. The mutation of *G6PD* was confirmed by Sanger sequencing (Figure 2A). No G6PD enzyme activity was detected in this patient, with a value of 0 U/g Hb. The G6PD enzyme activity of his mother was 3.5 U/g Hb. The mRNA level of G6PD was higher in the patient than in the healthy control and mother (Figure 2B). A comparative 3D structure of the *G6PD* p. R166C mutant showed that the mutation might affect the hydrophobic binding of the amino acid residues to other nearby residues (Figure 2C). As a consequence, the secondary structure of the G6PD protein would be influenced as well as the enzyme activity.

### Reactive oxygen species production by peripheral blood mononuclear cells

ROS production in the PBMCs from the patient and the mother was also tested. Similar to the neutrophil results, ROS production was obviously enhanced after stimulation in healthy PBMCs, whereas in the patient cells, no difference in fluorescence intensity was observed after stimulation. LPS stimulation slightly improved ROS production in PBMCs derived from the mother; however, this effect was not as strong as that in control cells (Figure 1B).

### Nuclear factor kappa-B activation in the cells

**FIGURE 3:**
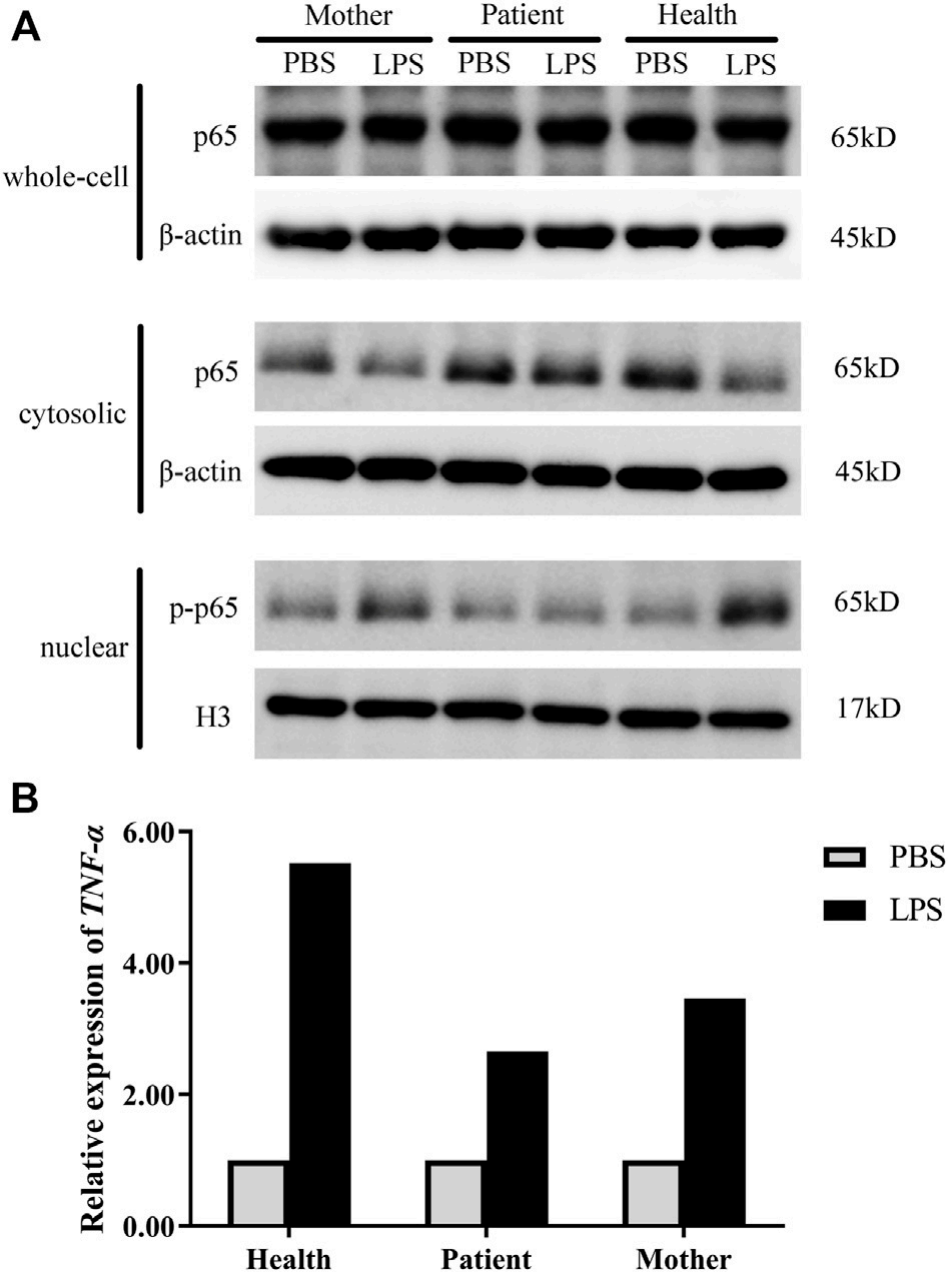
NF-κB activation in the patient. **(A)** The protein levels of p65/p-p65 in whole-cell lysates, cytosolic fractions, and nuclear extracts were measured by western blot analysis. **(B)** mRNA accumulation of the TNF-α gene in cells derived from healthy controls and the patient after LPS stimulation. PBS, unstimulated treatment; LPS, stimulated with LPS. Normalized target gene mRNA accumulation is reported relative to the mRNA accumulation in the PBS treatment of the healthy controls, which was set to 1.

The results showed that in the healthy control, the expression level of total p65 protein was almost equal in whole-cell lysates from the PBS and LPS treatments, and the p65 level was decreased in the cytoplasm, whereas phospho-p65 (p-p65) was increased in the nucleus after LPS stimulation, indicating normal initiation of the NF-κB signaling pathway. In the mother, p-p65 in the nucleus after LPS stimulation also increased but was slightly lower than that in the healthy control, whereas in the patient, the p-p65 level in the nucleus was significantly decreased. These results indicated that activation of NF-κB signaling was influenced in the patient and his mother (Figure 3A). We then detected the relative expression of TNF-α. As a consequence, the TNF-α level after LPS stimulation was 5.52 times greater than the unstimulated cells in the healthy control; in addition, it was 2.65 times greater in the patient and 3.64 times greater in his mother (Figure 3B).

### Nonrandom inactivation of the X chromosome in leukocytes and neutrophils

The double peaks of the fluorescence intensity curve for PMA-stimulated neutrophils from the mother represent two populations of neutrophils, similar to the X-CGD carrier. We speculate that this phenomenon, unusual decreased ROS production and low SI of the mother, was related to X chromosome inactivation. The mother’s results showed that 66.04% of the X chromosome carrying the mutated gene was inactivated in leukocytes, and 74.71% was inactivated in neutrophils (Table 2).

**TABLE 2:** Results of X chromosome inactivation detection.

Sample		Size	Hight (u)	Hight (d)	Inactivation (%)
control	leukocytes	281.8	1145	NA	—
		NA	NA	NA	—
	neutrophils	293.9	1922	NA	—
		NA	NA	NA	—
patient	leukocytes	296.3	2291	NA	—
		NA	NA	NA	—
	neutrophils	296.74	4334	NA	—
		NA	NA	NA	—
mother	leukocytes	287.66	2767	810	33.96
		296.18	2152	1225	66.04
	neutrophils	288.01	1349	945	25.29
		296.69	898	1858	74.71

u, undigested samples; d, enzyme-cut samples

### Literature review

**FIGURE 4:**
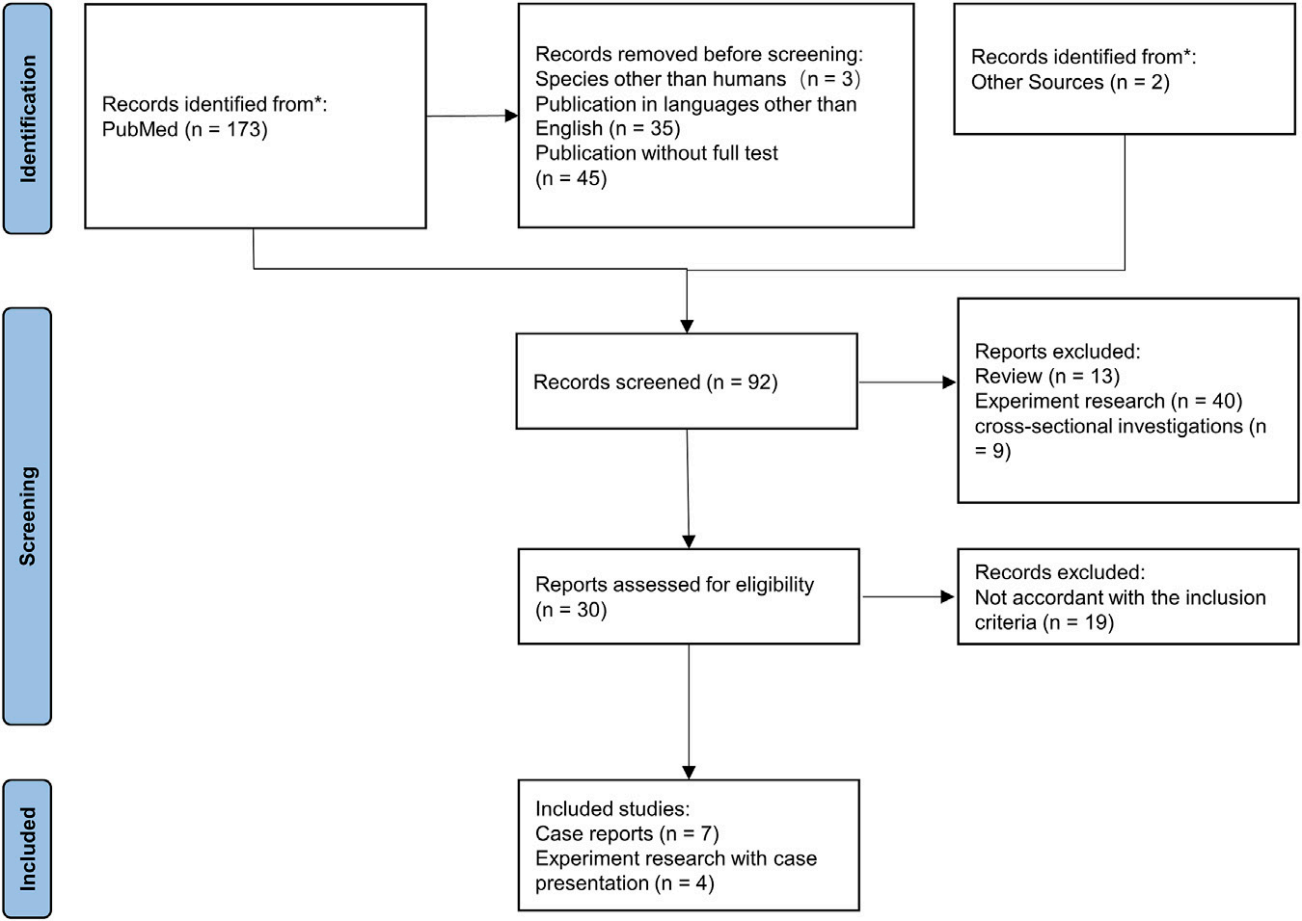
PRISMA flowchart for the systematic literature review. Inclusion and exclusion of references are shown.

**FIGURE 5:**
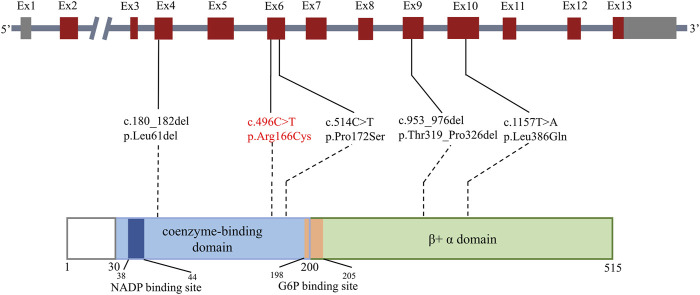
Mutations related to severe G6PD deficiency and potential neutrophil dysfunction. The bar above shows the distribution of reported gene mutations in the cDNA. The bar below shows the distribution of reported gene mutations in the protein. Exons are shown as thick numbered blocks, and introns are shown as thin lines. Noncoding regions are in gray. The mutation c.496 C>T in our study is in red. The long intron two is indicated by a diagonal double line.

Ultimately, 15 patients in 11 studies with severe G6PD deficiency and infections were reviewed in PubMed (Cooper et al., 1972; Gray et al., 1973; Vives Corrons et al., 1982; Roos et al., 1999; Rosa-Borges et al., 2001; Spolarics et al., 2001; Costa et al., 2002; van Bruggen et al., 2002; Khan et al., 2017; Siler et al., 2017; Thwe et al., 2020), which might be explained by neutrophil dysfunction. The PRISMA flow diagram is shown in Figure 4, and the data are summarized in Table 3. The majority of patients were male (13/15), but two females also had clinical manifestations. The median age of onset of infection was 4.5 years (range, 0–34 years). Patients presented with recurrent infections in childhood. The infection sites were mainly located in the respiratory tract and digestive tract and presented as pneumonia or bronchopneumonia (9/15), upper respiratory infection (3/15), or gastroenteritis and diarrhea (4/15). The most common bacteria included *Chromobacterium violaceum* (3/15) and *Serratia marcescens* (2/15). Two patients experienced *Bacillus* Calmette-Guérin (BCG) infection, and one patient experienced *Aspergillus* infection. Most patients could recover from recurrent infection after antibiotic treatment; however, two patients died due to severe sepsis. Five *G6PD* gene mutations were reported, including c.1157T>A, c.180_182del, c.514C>T, c.496C>T, and c.953_976del; the mutation c.496C>T resulted in amino acid changes, which is consistent with our study. Mutations were located in exon 4, exon 6, exon 9, and exon 10 (Figure 5). The G6PD activity in erythrocytes and granulocytes of patients was absent or low. The results of the nitroblue tetrazolium (NBT) reduction assay (8/15) and DHR oxidation assay (6/15) were available, which suggested a marked reduction in respiratory burst activity in most patients.

**TABLE 3:** Severe G6PD deficiency with recurrent infections in patients reported in the literature.

Patient ID	Age of onset (y)/sex	Clinical feature	Pathogen	Antibiotic therapy	G6PD activity		Neutrophil function tests		cDNA nucleotide substitution	Amino acid substitution	Refs
					Granulocytes	Erythrocyte (U/g Hb)	NBT	DHR			
P1	34 years/female	hemolytic anemia, leukemoid reaction, upper respiratory infections, urinary tract, bronchopneumonia, pneumonia, sepsis (died)	*Escherichia coli*, *Klebsiella*	sulfonamides, cephalothin, kanamycin, gentamicin	absent	low	no evidence of NBT reduction	ND	NA	NA	Cooper et al. (1972)
P2	10 years/male	acute intravascular haemolysis, haemoglobinuria, bronchopneumonia and anemia, cervical lymphadenitis, chronic granulomatous inflammation	NA	penicillin, sulphamethazine	0	0	positive cell<1%	ND	NA	NA	Gray et al. (1973)
P3	8 years/male	neonatal jaundice, infectious hepatitis, anemia, jaundice, upper respiratory infections, cervical node infection		cloxacilin							
P4	4 years/male	severe hemolytic anemia, bacterial pneumonia	NA	NA	0	0	severely decreased	ND	NA	NA	Vives Corrons et al. (1982)
P5	2years/male	sepsis (died)	*C. violaceum*	ampicillin, moxalactam, gentamicin, and lincomycin	ND	ND	ND	ND	NA	NA	Mamlok et al. (1987); Thwe et al. (2020)
P6	2 years/male	gastroenteritis, bacteremia	*Campylobacter* jejuni, *C. violaceum*	meropenem, levofloxacin, doxycycline	3.5% of the control	0	positive cell 4%	ND			
P7	NA/female	severe icterus, hemolysis, pneumonia, cystitis	Pneumococcus	NA	3.6 U/g protein in Leukocytes 5.2 U/10^10 cells in Neutrophils	0.1	NA	ND	c.514C>T	p. Pro172Ser	Roos et al. (1999)
P8	0.5 years/male	pneumonia, pleural effusion, septicemia, sinusitis, otitis media, stomatitis, skin suppurative lesions, diarrhea	NA	cotrimoxazole	ND	1.8–37	7.7% of the control	5.7% of the control	NA	NA	Rosa-Borges et al. (2001)
P9	15 years/male	fever, gastroenteritis, coughing, disseminated aspergillosis	Aspergillosis	amphotericin B, flucytosine, itraconazole	0	0.1	NA	NA	c.180-182delTCT	p. Leu61del	van Bruggen et al. (2002)
P10	3.5 years/male	high fever, coughing, pneumonia, anemia	*C. violaceum*	cephalosporin, meropenem, ciprofloxacin							
P11	5 years/male	neonatal jaundice, hemolysis, gastroenteritis, upper respiratory infections	NA	NA	0	0	ND	reduction in oxidative burst	c.953-976del	p. Thr319_Pro326del	Costa et al. (2002)
P12	10 years/male	tonsillitis, pneumonia	EBV, *Serratia marcescens*	co-trimoxazole	0	0	marked reduction in respiratory burst activity	marked reduction in respiratory burst activity	c.496C>T	p. Arg166Cys	Siler et al. (2017)
P13	6.5 years/male	otitis media, abscessing lymphadenitis	*Serratia marcescens*	co-trimoxazole							
P14	At birth/male	BCG adverse effect, tonsillitis, otitis media, pneumonia	*S. aureus*, BCG	NA	ND	NA	NA	low oxidative burst	c.1157T>A	p. Leu386GLn	Khan et al. (2017)
P15	At birth/male	BCG adverse effect, pneumonia									

y, year; BCG, Bacillus Calmette-Guérin; EBV, Epstein‒Barr virus; NBT, nitroblue tetrazolium; DHR, Dihydrorhodamine; NA, Not Available; ND, Not done

## Discussion

G6PD deficiency is widely recognized as a disease associated with hemolytic anemia, and its association with infection has not been fully appreciated. G6PD deficiency class I was included in PID in 2017 (Picard et al., 2018). Cases with severe G6PD deficiency and infections have rarely been reported; however, this figure may be an underestimate. A recently published study focused on infection in hospitalized patients with G6PD deficiency (Alrahmany et al., 2022). Only 620 of 3,334 registered G6PD-deficient patients did not have any microbiological cultures or hospitalization details. The incidence of G6PD deficiency in septic neonates was significantly higher than that in the control group (Zekavat et al., 2019); therefore, G6PD deficiency has been considered to be a risk factor for neonatal sepsis (Rostami-Far et al., 2016). It is necessary to focus on the clinical manifestations and immune function of patients with G6PD deficiency.

Similar to CGD, severe G6PD deficiency can manifest as recurrent infections. The age of onset was mostly in childhood and adolescence (14/15), emphasizing the need to identify these patients in early childhood. Patients had pneumonia, diarrhea, lymphadenitis, otitis media and even sepsis or cerebral infection. G6PD deficiency presents with recurrent bacterial and fungal infections. The susceptibility to pathogens includes *Staphylococcus, Streptococcus, Salmonella, Escherichia coli, Aspergillus, etc.* (Siler et al., 2017). This disease makes patients susceptible to *Mycobacterium* species (Khan et al., 2017). The child in this study also developed respiratory tract, digestive tract and skin infections, which were thought to be bacterial, fungal, or viral in etiology and had a definite history of EBV infection. We believe that similar to CGD, patients with severe G6PD deficiency may need to be given long-term prophylactic antibiotics after early diagnosis to avoid serious consequences of infection.

The lack of NADPH due to G6PD deficiency results in impaired synthesis of ROS. Respiratory burst, the robust ROS production by neutrophils, plays an essential role in host defense against pathogens. The G6PD-catalyzed reaction is the first step in the pentose phosphate pathway through which cells produce NADPH (Zhang et al., 2022). As the substrate of NADPH oxidase, NADPH is one of the important raw materials for ROS synthesis. It has been suggested that superoxide formation in phagocytes can be influenced when G6PD activity is less than 5% of normal values (Roos et al., 1999; Mason et al., 2007). The experimental results of this child and the mother were consistent with previous studies. In the neutrophils and PBMCs from the child and his mother, ROS production was significantly downregulated compared with that in healthy controls. Experiments on a variety of cells *in vitro* have shown that ROS can act as a second messenger to activate the NF-κB pathway, and directly stimulating cells with oxidants activated the pathway, whereas antioxidants inhibited pathway activity (Morris et al., 2022). The degree of NF-κB activation and TNF-α production in this patient were also affected, leading to the inability to remove pathogenic microorganisms. In clinical practice, respiratory burst function can be examined by the DHR assay as early as possible in patients with absent or very low G6PD enzyme function to evaluate and intervene early.

It is unclear whether the different locations of *G6PD* gene mutations are associated with neutrophil dysfunction. More than 300 *G6PD* mutations have been reported to date. However, only a few mutations have been identified in severe G6PD deficiency cases with abnormal granulocyte function (Roos et al., 1999; Costa et al., 2002; van Bruggen et al., 2002; Khan et al., 2017; Siler et al., 2017), including three point mutations and two deletion mutations, none of which were previously reported as hot spot variants for G6PD deficiency. These related mutations were distributed in exons 4, 6, 9, and 10 and were located in the coenzyme-binding domain and β+ α domain. Two G6PD patients with the hot spot variants Gly163Ser and Leu342Phe were also tested for respiratory burst function in this study, and both were normal (data not shown). Mutations c.180_182del, c.514C>T, and c.953_976del were reported to be associated with G6PD deficiency class I, and mutation c.496C>T was reported to be associated with class II(Luzzatto et al., 2020). Mutation c.496C>T was first described in an 11-year-old boy presenting with an acute hemolytic crisis due to the loss of G6PD enzyme activity (Moradkhani et al., 2012) following a viral infection, whose other susceptibility to infection was unknown. Three patients in a family with the same mutation site were reported in 2017 (Siler et al., 2017), presenting with tonsillitis, pneumonia, *Serratia marcescens* and EBV susceptibility. The p. R166C mutant might affect the hydrophobic binding of amino acid residues to other nearby residues, and it is likely that Cys166 may form a disulfide bridge with other Cys residues, which could disturb the local structure. Therefore, we consider that R166C is a common mutation site in severe G6PD deficiency related to infections.

Because the *G6PD* gene is located on the X chromosome, clinical symptoms are usually observed in hemizygous male patients. As early as 1999, a female patient with G6PD deficiency was reported to present with chronic nonspherocytic anemia, granulocyte dysfunction, and increased susceptibility to infections (Roos et al., 1999). A completely skewed X chromosome inactivation was further confirmed. Moreover, the degree of X chromosome inactivation may be different in whole blood and granulocytes (Manco et al., 2011). The DHR of the mother in our report was reduced, with an SI of 37. We further analyzed the ratio of chromosome inactivation and found that 74.71% of neutrophils expressed normal enzyme activity. A %DHR + value of greater than 20% in CGD carriers was not significantly associated with infection (Marciano et al., 2018). Therefore, this mother did not develop infections despite lower ROS production than healthy controls.

We first reported one individual child with severe G6PD deficiency and recurrent infections in China, deepening our understanding of the disease. The intervention was a random, convenience sample with sample size limitations. In addition to class I, patients with class II may also need to be alert to the risk of immunodeficiency. Although there was only one case of a patient described in this study, the experimental results were consistent with other similar studies. In addition to ROS production, we further performed NF-κB activation and TNF-α expression assays. We summarized the previously reported cases and mutation sites related to severe G6PD deficiency in the literature and expect more cases to be found in the future.

In conclusion, severe G6PD deficiency, which includes class II in addition to class I, can affect neutrophil function, resulting in a CGD-like phenotype. Gene analysis revealed that p. R166C was a common mutation site in unrelated families. For early diagnosis, the DHR assay is recommended to evaluate neutrophil function for patients with severe G6PD deficiency identified by neonatal screening, and antibiotics are recommended to prevent infection if necessary.

## Data Availability

The data presented in the study are deposited in the NCBI repository, accession number PRJNA888424.
